# Taxon ordering in phylogenetic trees by means of evolutionary algorithms

**DOI:** 10.1186/1756-0381-4-20

**Published:** 2011-07-01

**Authors:** Francesco Cerutti, Luigi Bertolotti, Tony L Goldberg, Mario Giacobini

**Affiliations:** 1 Department of Animal Production, Epidemiology and Ecology, Faculty of Veterinary Medicine, University of Torino, Via Leonardo da Vinci 44, 10095, Grugliasco (TO), Italy; 2 Molecular Biotechnology Center, University of Torino, Via Nizza 52, 10126, Torino, Italy; 3 Department of Pathobiological Sciences, School of Veterinary Medicine, University of Wisconsin-Madison, 1656 Linden Drive, Madison, Wisconsin, 53706, USA

## Abstract

**Background:**

In in a typical "left-to-right" phylogenetic tree, the vertical order of taxa is meaningless, as only the branch path between them reflects their degree of similarity. To make unresolved trees more informative, here we propose an innovative Evolutionary Algorithm (EA) method to search the best graphical representation of unresolved trees, in order to give a biological meaning to the vertical order of taxa.

**Methods:**

Starting from a West Nile virus phylogenetic tree, in a (1 + 1)-EA we evolved it by randomly rotating the internal nodes and selecting the tree with better fitness every generation. The fitness is a sum of genetic distances between the considered taxon and the *r *(radius) next taxa. After having set the radius to the best performance, we evolved the trees with (*λ *+ *μ*)-EAs to study the influence of population on the algorithm.

**Results:**

The (1 + 1)-EA consistently outperformed a random search, and better results were obtained setting the radius to 8. The (*λ *+ *μ*)-EAs performed as well as the (1 + 1), except the larger population (1000 + 1000).

**Conclusions:**

The trees after the evolution showed an improvement both of the fitness (based on a genetic distance matrix, then close taxa are actually genetically close), and of the biological interpretation. Samples collected in the same state or year moved close each other, making the tree easier to interpret. Biological relationships between samples are also easier to observe.

## Background

A central goal of evolutionary biology is to describe the "Tree of Life", inferring relationships among all living organisms. First appeared in the XIX century, trees were often used to describe relationships among organisms, but only Charles Darwin, in his revolutionary Origin of the Species [1], was the first to define them as evolutionary trees. Instead of using phenotypic characters, as Darwin did first, nowadays such trees are commonly built on genetic information and models of molecular evolution.

**Figure 1:**
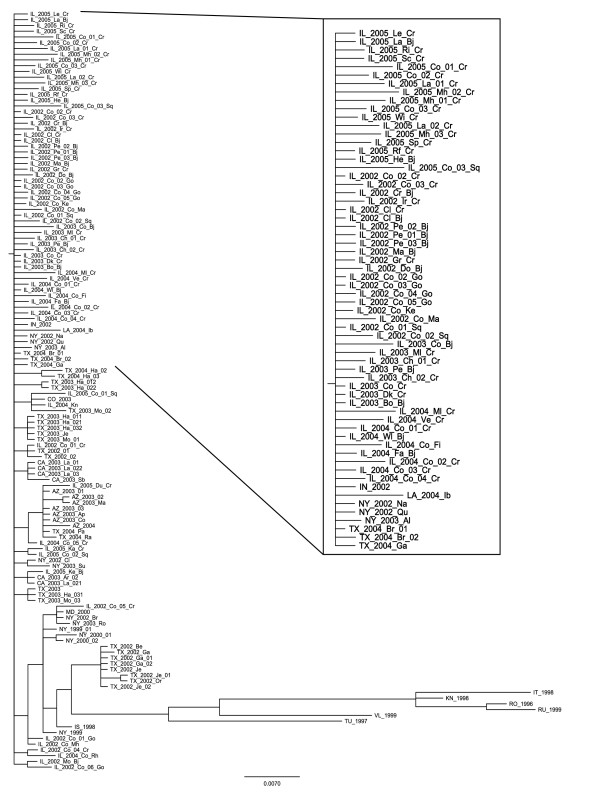
**WNV original tree**. WNV phylogenetic tree obtained by the Bayesian approach within MrBayes software and used as starting tree for all the algorithms performed in this work [12].

A phylogenetic tree is a mathematical structure to represent the evolutionary history of sequences or individuals. It consists of nodes connected by branches (or edges). The terminal nodes represent the "leaves" of the tree (or tips of the branches) and are also called taxa. Internal nodes represent ancestors, and can be connected to many branches; in this case the node is a politomy and represents either simultaneous divergence of descendants (hard politomy) or uncertainty about the phylogenetic relationship (soft politomy) [2,3]. Trees with many politomies are called unresolved trees, as they do not resolve the full history of evolution that they represent. The order in which the labels of the tips are drawn can differ without changing the meaning of the tree itself. This because in a tree the branches can be freely rotated without modifying the relationship among taxa [4,5]. The so called additive trees have branches containing information about the degree of difference between nodes, and they are used to show evolutionary features. In this case, information in the tree is contained in the branch direction, in the pattern of linkages between branches and nodes or, in other words, in its topology. Indeed in the representation commonly used by phylogenetic softwares, with the root on the left and the tips on the right (Figure 1), the order of taxa is meaningless, and the degree of similarity between taxa is only reflected by the branch path between them [5,6]. A second important feature of phylogenetic trees is the node's degree: in a fully resolved tree, all internal nodes have a degree equal to three, but, because of polytomies, trees may be hard to interpret and then being misinterpreted, assigning unfounded meaning to the proximity of taxa or clades. If the order of taxa on phylogenetic trees is flexible, ascribing biological meaning to it without altering the topology is possible [2,5].

A first approach to reorder the taxa according to a distance matrix was introduced by Moscato, Cotta and colleagues [7,8]. In these works, the authors both build new phylogenies and improve existing ones generated by Neighbor Joining and hypercleaning methods. They approached the problem as a minimum Hamiltonian path problem, and used memetic algorithms to find the "solution that minimizes the length of a path of distances between species" [8].

Here we propose a different approach belonging to the Evolutionary Algorithms (EAs) family to search a better graphical representation of an unresolved tree [9,10]. Given that each node can be freely rotated, one could group taxa with similar features, such as genetic similarity, geographic location or collection date, preserving the original topology. This approach could assist in drawing phylogenetic trees including more information, especially in highly unresolved trees.

Considering a tree with its own topology (either rooted or unrooted, and previously determined by means, for example, of neighbor joining, maximum likelihood, maximum parsimony, or Bayesian phylogenetics), the search space contains all the possible tree representations obtainable by node rotation. In this environment, the search problem would be to find the graphical representation that minimizes the distances between adjacent taxa. Such distances can be defined in separated matrices, obtained from different taxa features like genetic, temporal or geographic data.

The reason that moved us towards the application of a heuristic approach instead of an exhaustive search lies in the tree itself. Given a topology with *N *nodes, each with degree {*d*_1_, *d*_2, _..., *d_N _*} (the root having one branch), the space *S *of all possible trees would have the size:

In the case of a phylogenetic tree with 64 taxa, this dimension would range from |*S*| = 2^63 ^in the case of a completely resolved tree (containing 63 internal nodes all with degree 3), to |*S*| = 64! for a completely unresolved tree. The simplest approach to the problem would be a hill-climbing algorithm starting from the tree obtained by any of the available phylogenetic softwares, where the taxon order is often the same order of the sequences input alignment. This search would be computationally unfeasible in most cases, for the reason that, for each tentative solution *s*, the number of neighbors |*neigh_s_*| to be generated *(all the trees with same topology, but only one node being rotated) *and evaluated would be:

where {*d*_1_, *d*_2_, ..., *d_N_*} are the degrees of the *N *internal nodes of the tree topology. Considering the previous case of the tree with 64 clades, this value would range from |*neigh_s_| *= 64 to |*neigh_s_*| = (64 × 63) = 2 = 2, 016.

In order to find the optimal solution to our problem, we chose to use (*λ *+ *μ*)-EAs, where *λ *is the parent population from which the child population *μ *is generated. In general, a (*λ *+ *μ*)-EA can be seen as a natural selection process (being inspired by the biological world), where the *λ *parents mate, generate *μ *children (different from them), and the fittest to the environment (i.e. the problem to be solved) survives and mate again. The *λ *parents for the next generation are given by the best (*λ *+ *μ*) individuals. Generation after generation, the population itself is selected to fit the environment (i.e. to solve the problem).

As first approach, we decided to use a (1 + 1)-EA, that could be interpreted as first-ascent hill climber. In each generation a new tree is created by random swap between two branches connected to the same node and evaluated according to a fitness criterion. Defined the *radius *(*r*) as the number of taxa adjacent to a considered taxon, the fitness of each tree is evaluated as the sum of all the distances between each node and the closest *r *tips according to the genetic distance matrix. The fittest tree (i.e. that tree with a taxon order that minimizes the distances) survives the next generation. After having explored the parameter *r *in the algorithm and compared the results with random searches, we investigated the influence of population size with different combinations of *λ *and *μ *values [11]. In this work we only considered a genetic distance matrix applied to the phylogenetic tree of West Nile virus (WNV) presented by Bertolotti and colleagues and thus exhaustedly explained in [12].

## Results and Discussion

The two experimental phases described at the end of the previous section are here described and discussed.

### (1 + 1)-Evolutionary Algorithm

Using the tree shown in Figure 1 as starting point for our search, we performed 50 runs of the (1 + 1)-EA for each radius *r *= 1, 4, 8, 32 considered for the fitness evaluation. The radius is meant as the number of close taxa considered during the fitness evaluation of a single taxon. Thus, for each taxon of the tree, the sum of the distances between it and the *r *closest taxa is considered as partial fitness. The resulting sum is the fitness of the tree according to the given distances and to the *r *value. We arbitrarily chose 4 values to test the radius, as the evaluation with *r *= [1, 131] would be meaningless: increasing the value of *r *leads to a flattening of the fitness, as the distance between each taxon and the *r *next taxa would likely be similar if not equal.

For the comparison of the fitness improvement between the different radii, we calculated the fitness of each tree as its relative fitness improvement (obtained as ratio between fitness of the considered tree and fitness of the original starting tree). Then we ranked each run as Gold, Silver and Bronze, corresponding to 0.80, 0.85 and 0.90 relative fitness improvement, respectively (the lowest fitness represents the best improvement). Statistics on this classification is reported in Table 1.

**Table 1:** Relative fitness improvement using (1 + 1)-EA

	*r *= 1		*r *= 4		*r *= 8		*r *= 32	
	hits	generation	hits	generation	hits	generation	hits	generation
Gold	0	N/A	38	89, 349_±7,906.3_	50	39, 092_±4,770.1_	0	N/A
Silver	26	93, 243_±7,178.2_	38	67, 230_±9,409.3_	50	2, 035_±160.0_	0	N/A
Bronze	27	61, 810_±9,053.5_	39	41, 309_±6,638.4_	50	714_±83.4_	50	3, 239_±195.7_

For each fitness radius the number of runs (hits) over the 50 executed is shown, as well as the mean generation together with its standard error at which the target was found.

With *r *= 1, no tree in Gold position was found, but more than half of the 50 runs reached both Silver and Bronze ranks. For *r *= 4 more than half of the runs reached the Gold position. Apparently, better results were obtained with *r *= 8, as all the 50 runs reached the Gold class. When *r *= 32 was used in the fitness evaluation, all the runs improved only until Bronze rank. Trees at *r *= 8 reached the Bronze class always in less than 1, 000 generations, while the other radii obtained this improvement only after 40, 000 generations in average.

**Figure 2:**
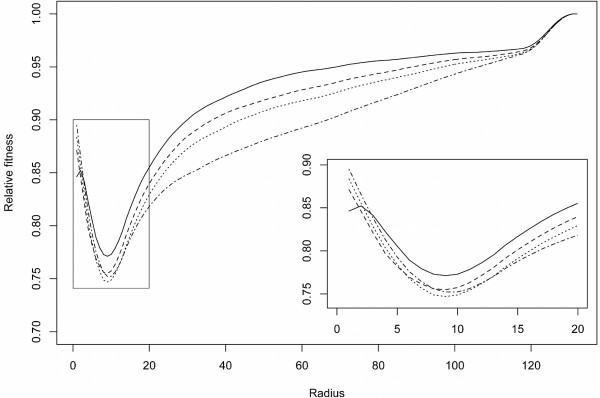
**Evaluation with all the possible radii**. Median fitness improvements evaluated with *r *∈ [1, 131] for the runs evolved using the 4 different radii (solid line: *r *= 1; dashed line: *r *= 1; dotted line: *r *= 8; dash-dotted line: *r *= 32). Box area highlights the radius range [1, 20].

In order to compare the trees obtained using different radii, all trees were evaluated with *r *ranging from 1 up to 131 (the total number of the taxa). Figure 2 shows the median fitness improvements evaluated with *r *∈ [1, 131] for the runs evolved using the 4 different radii (with a focus on the range [1, 20]). It can be observed that the radii 8, 9, and 10 correspond to the region where all the trees showed the largest relative fitness improvement. This evidence, together with the aforementioned ranking analysis, suggests these radii as those exhibiting the best evolvability, therefore in the following (*λ *+ *μ*)-EAs we performed the fitness evaluation with *r *= 8 only.

To compare the relative fitness improvement using a different unit, we evaluated all the final trees with *r *= 75, a value that is well above the one used in the search algorithm and that still allows discrimination between the different curves in Figure 2.

Statistical analysis of relative fitness improvements of the 4 different groups evaluated at *r *= 75 showed a significant difference between them (Kruskal-Wallis rank sum test *p <*0.001). Moreover, these data highlighted a significant ordering among the groups, having *r *= 1 worse than *r *= 4, *r *= 4 worse than *r *= 8, and *r *= 8 worse than *r *= 32 (all pairwise Wilcoxon rank sum test *p <*0.001).

To justify the use of our fairly complex heuristic search method, we performed for comparison a random search (RS) algorithm with the same number of evaluations (n = 200000). Briefly, our (1 + 1)-EA found trees with relative fitness improvement of 0.8344 (for *r *= 1), 0*:*7943 (for *r *= 4), 0.7478 (for *r *= 8), and 0*:*8511 (for *r *= 32), whereas RS method found trees evaluated at 0.9065 (for *r *= 1), 0.8206 (for *r *= 4), 0.7699 (for *r *= 8), and 0.8878 (for *r *= 32). Wilcoxon rank sum test performed between EA and RS returned *p *≪ 0.001 for each *r *values, showing that the evolutionary search methods consistently outperform random search with a strong statistical support.

### (*λ *+ *μ*)-Evolutionary Algorithms

As in the (1 + 1)-EA study, we performed 50 runs for each parameter of the population study. In details, we studied two different parameters for (1 + *μ*)-EA, where *μ *= (5, 10), and five combinations of parameters for (*λ *+ *μ*)-EAs: (5 + 5), (5 + 10), (10 + 10), (50 + 50) and (1000 + 1000). As starting tree, the same WNV original tree was used and its fitness at *r *= 8 was considered as starting fitness for the computation of relative fitness improvement (final tree fitness/original tree fitness).

**Figure 3:**
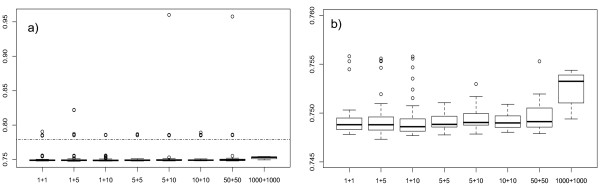
**Relative fitness improvement by (*λ *+ *μ*)-EAs**. Boxplot representing the relative final fitness values for the (*λ *+ *μ*)-EAs (a) and magnified in the range [0.745, 0.76] (b). The dotted line in a) is the threshold used to evaluate the computational effort and set at 0.78.

The best result, or in other words the best fitness improvement, was obtained by the (1 + 5)-EA, giving a relative improvement of 0.75. Nevertheless the Wilcoxon Rank Sum test performed between EAs returned not significant differences except in the case of the (1000 + 1000)-EA, that returned *p - value *≪ 0.01. Boxplots in Figure 3 highlight how (1000 + 1000)-EA has a lower performance in finding better solutions compared to the other populations (Figure 3b), although the distribution of its final fitness values is the only one in which all runs cross the threshold of relative improvement of 0.78 (Figure 3a). Hit rates reported in Table 2 also underline that all (1000 + 1000)-EAs cross the threshold. The other (*λ *+ *μ*) combinations instead succeeded only in between 90% and 94% of the runs. This threshold was set to compare the convergence speed of the different populations and its value was arbitrarily chosen in the range [0.757, 0.780], as a change in this value does not affect the conclusion. For convergence speed we mean the number of evaluations needed to cross this threshold, and these values are reported in Table 2. The convergence speed of the (1000 + 1000)-EA is statistically lower than the other EAs (p-values ≪ 0.01 in Wilcoxon Rank Sum tests), resulting in the best computational performances but in the worse solution by one order of magnitude (Figure 3). Additionally the (50 + 50)-EA convergence speed is statistically better than (1 + 1), (5 + 10) and (10 + 10)-EAs.

**Table 2:** Hit rate and Convergence speed for (*λ *+ *μ*)-EAs at *r *= 8

	1+1	1+5	1+10	5+5	5+10	10+10	50+50	1000+1000
Hit Rate	0.92	0.90	0.94	0.94	0.90	0.90	0.87	1.00
Conv. speed	60917.24	51712.44	59373.83	53641.17	69464.67	71369.56	42705.77	4800.00
	6810.97	6740.09	7168.53	6655.89	8098.49	8597.38	9149.91	304.75

Hit rate and convergence speed are reported in the table. Hit rate is the rate of runs that had a relative improvement greater than 0.78. The convergence speed is normalized to the number of evaluations and represents the mean generation (second row) and standard error (third row) that crosses the threshold 0.78

### Biological interpretation of the results

**Figure 4:**
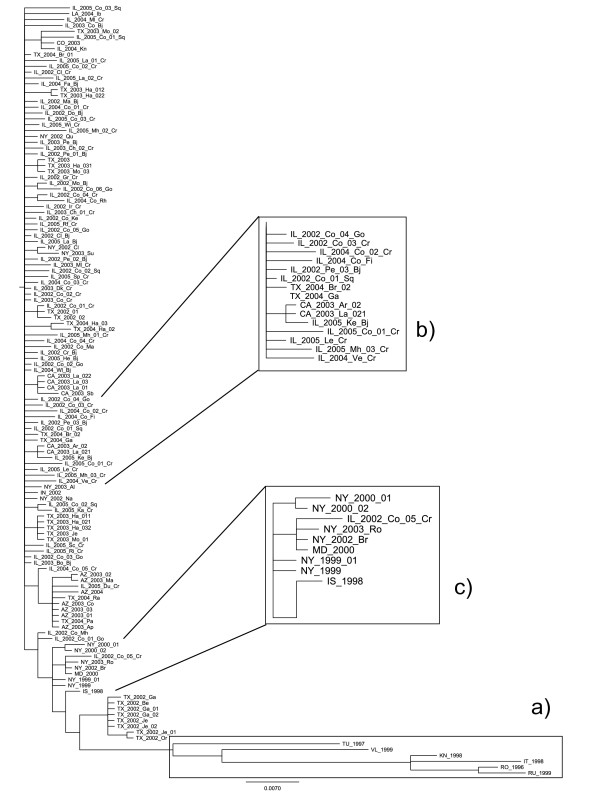
**(*λ *+ *μ*)-EA best tree**. The new graphic representation of the best result among final trees obtained by the (*λ *+ *μ*)-EA, (1 + 5) in particular. Biologically meaningful improvements in the vertical order of taxa can be seen, involving samples collected in the same year or state (magnified in b), or the clade containing European strains was moved to the bottom of the tree (a), and the relationship between the NY99 and Israel98 samples is highlighted (c).

The best tree among all the runs was obtained by the (1 + 5)-EA and it is shown in Figure 4. From the biological point of view, the tree has undergone some improvement for the readability of taxon order. At first sight, the most notable change is the long branch containing the European strains of WNV moved to the bottom of the tree from its previous middle position (Figure 4a). Moreover, many samples collected in the same state are now close to each other (Figure 4b). The algorithm has also moved closer those tips representing samples collected in the same year (Figure 4b). Finally, as demonstrated by Lanciotti and colleagues in [13], the strain of the first WNV epidemic in USA, in New York in 1999, is next to the strain associated with an epidemic in Israel in 1998, which arrived in the new world and likely sparked the North American WNV epidemic. In our final tree this relationship is highlighted (Figure 4c).

## Conclusions

Since unresolved trees are hard to interpret, we studied the behavior of different parameters in (*λ *+ *μ*)-EAs to explore the search space of all the solutions to our problem: making unresolved trees more meaningful. To do this we used an already published tree as starting point [12], and a matrix of genetic distances among samples, corrected with the best fit molecular substitution model, in the fitness computation. First, we investigated the influence of different radius values (*r *= 1, 4, 8, 32) in evaluating the fitness in the (1 + 1)-EA. The most informative results were obtained using *r *= 8, though all the considered radii performed better than the random search. Although, as expected, trees evolved using *r *= 32 showed improved fitness with respect to the other groups, the computational time needed to evolve these trees is long. Statistical analyses of the experimental data, together with the observations described above, suggest that a value for the radius in the fitness evaluation *r *= 8 might generally strike an acceptable balance between computational intensity and accuracy.

After the identification of an efficient radius, we investigated the influence of population sizes in (*λ *+ *μ*)-EAs. All the combinations of *λ *and *μ *values returned solid results for the improvement in the graphical representation of the tree, adding important information to the vertical reading direction, although no differences in performance were observed for most of them. In fact, only the (1000 + 1000)-EA returned worse fitness of the final trees when compared to the other populations, but still better than the original tree. However, our experiments showed a significant gain in fitness values of one order of magnitude by this large population EA.

In a biological perspective, the best tree obtained in all the runs was improved by the (1 + 5)-EA. This outcome allows an easier interpretation of the tree, especially in those highly unresolved ones, with many siblings deriving from the same common ancestor. As previously discussed, in the improved tree, genetically more similar taxa are collected in a close spatiotemporal dimension and are now closer than in the original tree. This suggests that the use of genetic closeness between samples to improve the order of taxa in the tree somehow reflects the spatiotemporal relationship between them.

This work represents the first try to develop a new method to add a meaning to the order of taxa in a phylogenetic tree and it needs to be further investigated. So far we explored the dimension of the algorithm, studying the influence of the parameters on the performance during the process of selecting best trees. Next steps will be applying our method on different models analyzing different trees and different distances (e.g. geographic, temporal, serological) to test the algorithm in varying conditions.

## Methods

The two algorithms applied in the article are here described and discussed, together with the phylogenetic tree used to validate the new technique, and the statistical methods employed to analyze the experimental results.

### (1 + 1)-Evolutionary Algorithm

As starting tree for the algorithm, an original tree obtained by any available software for phylogenetic inference with any method (neighbor joining, maximum likelihood, maximum parsimony, Bayesian phylogenetics) is suitable. Starting from the original tree, in each generation a new tree is created by applying a random swap between two taxa connected to the same node in the tree. The fitness of the new tree is evaluated as sum of all the distances between each node and the closest *r *tips according to the genetic distances matrix (*r *being the radius in the fitness evaluation). If the fitness of the new tree is better than the one of the tree in the previous generation, the new tree replaces the old one, and the search procedure continues. If not, the old tree is retained. This process is iterated for 200000 generations, resulting in the creation and evaluation of 200000 new tentative solutions.

When evaluating the fitness of a tentative solution, the most straightforward method would be to sum up, for all taxa in the tree, the distance between the taxon under consideration and the two taxa next to it, i.e. taxa at radius *r *= 1. However, in order to have a wider overview to the taxa closeness, we tested different values of the radius in the fitness evaluation process. For each *r *value, 50 independent runs were performed. To justify the use of our heuristic search method, we performed for comparison a random search with the same number of evaluations (n = 200000) of trees generated by random swap of taxa, without memory of the previous generations.

### (*λ *+ *μ*)-Evolutionary Algorithm

A first exploration of simple population EAs was performed, where *λ *= 1 is the starting tree to improve, *μ *= 5, 10 and *r *= 8. Then, we evaluated the performances of the EAs when *λ *= *μ *∈ {5, 10, 50, 1000}.

When *λ >*1, *λ *- 1 trees generated by random swap are added to the original tree in the initial generation. In details, we tested the following populations: (1 + 5), (1 + 10), (5 + 5), (5 + 10), (10 + 10), (50 + 50) and (1000 + 1000).

The next generation of *λ *parents is selected by performing *μ *tournaments between couples chosen by random sampling with reintroduction among the *λ *+ *μ *individuals, selecting the best *μ *ones. In this way the best individual can potentially be selected more than once, without excluding the other tentative solutions. To have a fair comparison between algorithms with different population sizes, we set a maximum generation limit so that the number of new tentative solutions that each EA evaluates is 200000. For *μ *= 5, 10, 50, 1000 this results in 40000, 20000, 4000, and 200 generations, respectively. For each parameter combination, 50 independent runs each were performed.

### Experimental validation on WNV phylogenetic tree

WNV is a single stranded, positive-sense RNA virus member of the Japanese encephalitis serocomplex (*Flaviviridae*; *Flavivirus*) that is transmitted primarily through the bite of infected mosquitoes. Because of the recent introduction into North America, it has been possible to study the phylogenesis and the evolution of the virus. Such studies of the virus in North America reported highly unresolved trees [12,14,15]. In these cases, information on genetic, spatial or temporal clustering was not apparent, so that population substructure was investigated using different approaches.

In this study we used the tree presented by Bertolotti and colleagues in 2007, built using MrBayes software [16,17] (Figure 1).

The original tree has a total of 132 taxa and 28 internal nodes. The root node has 76 branches, among which 62 were directly connected to terminal taxa (as magnified in Figure 1), pointing out that this part of tree is highly unresolved. Relating to the previous formulas, the resulting search space is |*S*| = 1.749 × 10^137^, and every tree has 2, 975 neighbors, an area too large to be explored with a exhaustive search.

To calculate the fitness of each tentative solution, we used the matrix of genetic distances among samples, corrected with the best fit molecular substitution model (GTR+ Γ +I) [5,18].

### Statistical analysis

The data obtained by the runs were tested with the Shapiro-Wilk test to determine if the samples come from a normal distribution. As the test suggested a non-normal distribution, we applied the Kruskal-Wallis test for testing equality of population medians among groups. Finally we performed the Wilcoxon rank-sum test to study the variability of the results. All the tests were performed in R software [19].

### Computational performance

The effective code of the algorithms was written in R language, using the package 'ape' [20]. The runs were performed on the cluster IBM-BCX available at the Supercomputing Group of the CINECA Systems & Tecnologies Department at the time of the work.

## Competing interests

The authors declare that they have no competing interests.

## Authors' contributions

FC implemented the computational model, carried out the simulations, and participated in the interpretation of the results. LB conceived the design of the study, implemented the computational model, and participated in the interpretation of the results. TLG conceived the design of the study and participated in the interpretation of the results. MG conceived the design of the study, participated in the interpretation of the results, and coordinated the participants' contributions. All authors equally participated in writing the manuscript and approved it.
